# Oral Chinese Herbal Medicine on Immune Responses During Coronavirus Disease 2019: A Systematic Review and Meta-Analysis

**DOI:** 10.3389/fmed.2021.685734

**Published:** 2022-01-21

**Authors:** Shihua Shi, Fei Wang, Huan Yao, Shuo Kou, Weihao Li, Bonan Chen, Yongcan Wu, Xiaomin Wang, Caixia Pei, Demei Huang, Yilan Wang, Pan Zhang, Yacong He, Zhenxing Wang

**Affiliations:** ^1^Department of Geriatric, Hospital of Chengdu University of Traditional Chinese Medicine, Chengdu, China; ^2^Department of Rheumatology, Hospital of Chengdu University of Traditional Chinese Medicine, Chengdu, China; ^3^School of Public Health, Zhejiang Chinese Medical University, Hangzhou, China; ^4^Division of Cardiology, West China Hospital, Sichuan University, Chengdu, China; ^5^Department of Anatomical and Cellular Pathology, State Key Laboratory of Translational Oncology, Prince of Wales Hospital, The Chinese University of Hong Kong, Hong Kong, Hong Kong SAR, China; ^6^Acupuncture and Tuina School, Chengdu University of Traditional Chinese Medicine, Chengdu, China

**Keywords:** coronavirus disease 2019, lymphocytes, CD4—CD8 ratio, “medicine, Chinese traditional”, mortality, meta-analysis

## Abstract

**Background:**

Cellular immune responses including lymphocyte functions and immune effector cells are critical for the control of coronavirus infection. Chinese herbal medicine (CHM) potentially has a therapeutic effect for treatment of coronavirus disease 2019 (COVID-19). Nevertheless, there are limited clinical practice suggestions on immunogenicity of the CHM against SARS-CoV-2. To assess the effect of oral CHM on immunogenicity and whether oral CHM improves the clinical parameters through the immunity profile during COVID-19, we performed the present study.

**Methods:**

For this systematic review and meta-analysis, 11 databases were searched for relevant studies assessing oral CHM for COVID-19 on November 20, 2020 (updated March 9, 2021). Primary outcomes mainly included immunity profiles. Secondary outcomes included all-cause mortality; the remission time of fever, cough, chest tightness, and fatigue. The random effect was used to estimate the heterogeneity of the studies. Summary relative risks, weight mean difference and standardized mean difference were measured with 95% confidence intervals. Modified Jadad scale and Newcastle-Ottawa Scale were used to assess the risk of bias of randomized controlled trials (RCTs) and observational studies, respectively. The certainty of evidence was evaluated using the GRADE approach.

**Results:**

We analyzed findings from 3,145 patients in 30 eligible studies. Compared with routine treatment, oral CHM, as an adjuvant medicine, improved lymphocyte counts, CD4^+^, and CD4^+^/CD8^+^ ratio with low quality of evidence; improved CD3^+^ with moderate quality of evidence; and reduced TNF-α with low certainty of evidence. Besides, oral CHM, as an adjuvant medicine reduced the time to clinical symptoms remission with a lower risk of all-cause mortality, compared with routine treatment alone.

**Conclusion:**

CHM may be recommended as an adjuvant immunotherapy for disease modification and symptom relief in COVID-19 treatment. However, large RCTs objectively assessing the efficacy of CHM on immune responses in COVID-19 are needed to confirm our findings.

## Introduction

Coronavirus disease 2019 (COVID-19) caused by severe acute respiratory syndrome coronavirus 2 (SARS-CoV-2), contributed to the collapse of the immune system and has become a devastating pandemic with substantial mortality (1, 2). SARS-CoV-2, relevant to cytokine storm (a hyper-inflammatory response), stimulated the apoptosis of T-cell, causing the abnormal response of antiviral T-cell (1). Virus particles spread through the respiratory mucosa and infected ciliated bronchial epithelial cells, inducing a cytokine storm that caused changes in lymphocyte counts, CD3 T-lymphocyte counts, CD4 T-lymphocyte counts, CD8 T-lymphocyte counts, CD4^+^/CD8^+^ ratio, and leukocyte count in the body (2). Cellular immune responses consisting of CD3^+^ T, CD4^+^ T, and CD8^+^ T cells response underlying immunopathogenesis in COVID-19, which are critical for the control of coronavirus infection (1, 3). Immune dysfunction in COVID-19 patients has been attributed to pro-inflammatory cytokines including Tumor necrosis factor-α (TNF-α) and interleukin-6 (IL-6) (4). The specific cellular immunity and cytokine storm are associated with worsening of symptoms and the promotion of lung damage (5). Nevertheless, there are limited data on the associated immunological profile and the clinical parameters in COVID-19 treatment.

With the rise of “WE” medicine, proposed by Prof. Yung-Chi Cheng' team in Yale University, which might change human history, is a melding of Western medicine—focused on microscopic and single-disease targets—and Eastern medicine, exemplified by traditional Chinese therapies (6), the historical use of Chinese herbal medicine (CHM), the herbal agents or materials originated from botanical herbal products, animal, or mineral sources (7), for epidemic diseases has been captured attention in modern times. Given that the severe acute respiratory syndrome (SARS) virus is similar to coronavirus and CHM has successfully treated it (8), CHM potentially has a therapeutic effect for treatment of COVID-19. CHM was believed to possess immunopharmaceutical effects by modulating lymphocyte functions and immune effector cells during COVID-19, based on absorption, distribution, metabolism and excretion evaluation, target prediction, network construction and functional bioinformatics analyses (9). The most common formulas utilized in COVID-19 treatments were “3-drugs-3-formulas” with multi-component and multitarget characteristics (10), and the most common herbs used in COVID-19 were Baical Skullcap Root (Huangqin, Radix Scutellariae Baicalensis) and Liquoric Root (Gancao, Radix Glycyrrhizae), which were also included in YIV-906 (PHY906), a safe prescription drug that inhibits several inflammatory processes with activation of innate and adaptive immunity, and would probably be the first Chinese medicine approved as an FDA prescription drug (6, 11). Specifically, Baicalin, the active component of Baical Skullcap Root with antiviral and antibacterial effects, can inhibite inflammatory factors IL-6 and IL-1β, and activate the MAKP and NF-κB signaling pathways to induce IL-17 production, aiding the immune system (11). According to a preclinical study of baicalein on the treatment of COVID-19, Baicalein, which was also the active compound of Baical Skullcap Root with broad anti-virus effects, inhibited cell damage induced by SARS-CoV-2, inhibited the replication of the virus, and relieved the lesions of lung tissue in hACE2 transgenic mice infected with SARS-CoV-2 (12).

Compared to most of the affected countries in Europe and other continent, relying solely on routine treatments, namely, conventional western medicine including antiviral medications, antibacterial medications, steroids, symptomatic control, and supportive care (13), Asia and in particular, China, has adequate documentation of outcomes melding of Western medicine and eastern medicine in COVID-19 management. Although some work has been aimed at elucidating the improvement in CT scan, reverse transcriptase-polymerase chain reaction (RT-PCR) negativity rate, as well as the lower rate of adverse effects of COVID-19 patients treated with CHM, compared with routine treatment based on western medicine (14), there are limited clinical practice suggestions on the immunogenicity of oral CHM against SARS-CoV-2. It is unknown whether a combination of CHM interventions can improve the control of COVID-19 outbreak through regulating immunological profile in clinic, based on evidence-based medicine, though CHM, the natural immune boosters, might exert better immune enhancer activity compared with routine treatment alone based on the emerging studies (15–17).

Immunotherapeutic attempts against 2019-nCoV like vaccine and Immunoglobulin therapy have been the hot topic of COVID-19 researches recently (18). Given that CHM, used widely in COVID-19, has been considered as immunotherapy in many diseases (19), while whether CHM could be an immunotherapeutic strategy during COVID-19 has not been well studied with definitive results, we performed this systematic review and meta-analysis to evaluate the potential effect of CHM on immune related profile compared with routine treatment during COVID-19, and to investigate whether the clinical parameters were improved through the immunity profile based on randomized controlled trials (RCTs).

## Methods

### Protocol and Registration

The present study was conducted following the Preferred Reporting Items for Systematic Reviews and Meta-Analyses guidelines strictly and previously published in the International Prospective Register of Systematic Reviews database (ID: CRD42020214495).

### Literature Searches

A comprehensive searching of bibliographic and grey literature sources, including PubMed, Cochrane Library, Web of science, Science Direct, Scopus, Google Scholar, Embase, ProQuest, China Science and Technology Journal Database (VIP), China National Knowledge Infrastructure, WANFANG DATA, WHO covid-19 website, and Centers for Disease Control and Prevention COVID-19 websites of the US and China was performed as of November 20, 2020 (updated March 9, 2021) without language restriction. We also scrutinized the bibliographies of eligible studies and relevant review articles. We tried to contact with study authors to identify additional studies. The Medical Subject Headings (MeSH), free text and relevant terms of COVID-19 and CHM were applied in our search strategies, and could be found in Supplementary Materials.

### Study Identification and Outcomes

Pairs of reviewers (S.S. and H.Y) screened the titles, abstracts and full-text of candidate articles independently to assess eligibility. Discrepancies were resolved by discussion, and disagreement would be resolved by a third researcher (F. W.). RCTs were the optimal study design, but if the number of relevant RCTs on outcomes was less than three, observational studies with a control arm (routine treatment without CHM), including prospective or retrospective case series, or cohort studies, were considered for inclusion, considering the urgent need to respond to the COVID-19 pandemic. Thus, the studies were eligible if they were human RCTs, or observational studies including prospective or retrospective case series, or cohort studies, with a control arm treated with routine treatment like α-interferon, Ribavirin, Arbidol hydrochloride, Chloroquine phosphate, corticosteroids, respiratory support, and symptomatic treatment (20), without CHM; performed among COVID-19 patients; evaluated the effect of oral CHM as an adjuvant based on “WE” medicine on the outcomes of interest without limitation on dosage forms. The PICOS (patient, intervention, comparison, outcome, and study design) for study selection was shown in Table 1.

**Table 1:** PICOS for study selection.

**Parameters**	**Descriptions**
P	Patients with coronavirus disease 2019
I	Oral Chinese herbal medicine plus routine treatment
C	Routine treatment without Chinese herbal medicine
O	Lymphocyte count, CD4^+^, CD8^+^, CD4^+^/CD8^+^ ratio, CD3^+^, IL-6, TNF-α, leukocyte count, all-cause mortality, the time to the remission of fever, the time to cough remission, the time to the remission of chest tightness, time to fatigue remission.
S	Randomized controlled trials (if not available, observational studies)

*P, Patient; I, Intervention; C, Comparison; O, Outcome; S, Setting*.

The primary outcomes of the present study included lymphocyte counts, CD4^+^, CD8^+^, CD4^+^/CD8^+^ ratio, CD3^+^, Leukocyte counts, TNF-α, and IL-6. The secondary outcomes included all-cause mortality, the time to remission of clinical symptoms including cough, fever, fatigue, and chest tightness. Furthermore, the studies were excluded if they did not contain eligible comparators; lacked a control arm; included CHM in control arm (routine treatment); did not study the outcomes of interest; were *in vitro* or *in vivo* studies; were not related to COVID-19. Although suspected COVID-19 were closely related with SARS-CoV-2, the results may not be directly extrapolated to COVID-19 patients. For this reason, we excluded the studies of suspected and probable COVID-19.

### Data Extraction and Quality Appraisal

Pertinent information was independently extracted and then crosschecked by pairs of investigators (S.S. and Y.W.) using a data collection form that included the characteristics of trial and population, and outcomes of interest. Two assessors (W.L. and S.C) used the modified Jadad scale to assess the methodological quality of inclusive RCTs (21). The studies were considered to be of high quality if the modified Jadad scores were equal to or greater than 4 (21, 22). The quality assessment of cohort studies was performed by two review authors (S.S. and S.C) utilizing the New-castle Ottawa Scale (NOS) (23). Discrepancies were solved by consultation or adjudication by the corresponding author (Z. W.).

### Statistical Analysis and Certainty of the Evidence

Risk ratio (RR) was calculated for dichotomous data; weight mean difference (WMD) or standardized mean difference (SMD) was calculated for continuous variables, using 95% confidence interval (CI), with a *I*^2^ <25%, 26–50%, and > 50% assumed to indicate low, moderate, and significant degrees of heterogeneity, respectively. Random effects model was used to estimate the heterogeneity of the studies. Subgroup analysis was performed for study design if observational studies were included, because substantial variation between studies on study design was expected. Sensitivity analysis was carried out by removing data of each study from the pool to explore the robustness of the results. Potential publication bias was assessed in the outcome with the largest number of studies, using visual inspection of funnel plots. Meta-analysis was performed using Review manager software (version 5.4). The certainty of evidence was evaluated with the grading of recommendations assessment, development, and evaluation (GRADE) approach. Tow reviewers (S.S. and S.C.) evaluated the certainty of evidence with an independent third-party acted as an arbiter (P.Z.).

## Results

### Flow of the Included Study

**Figure 1:**
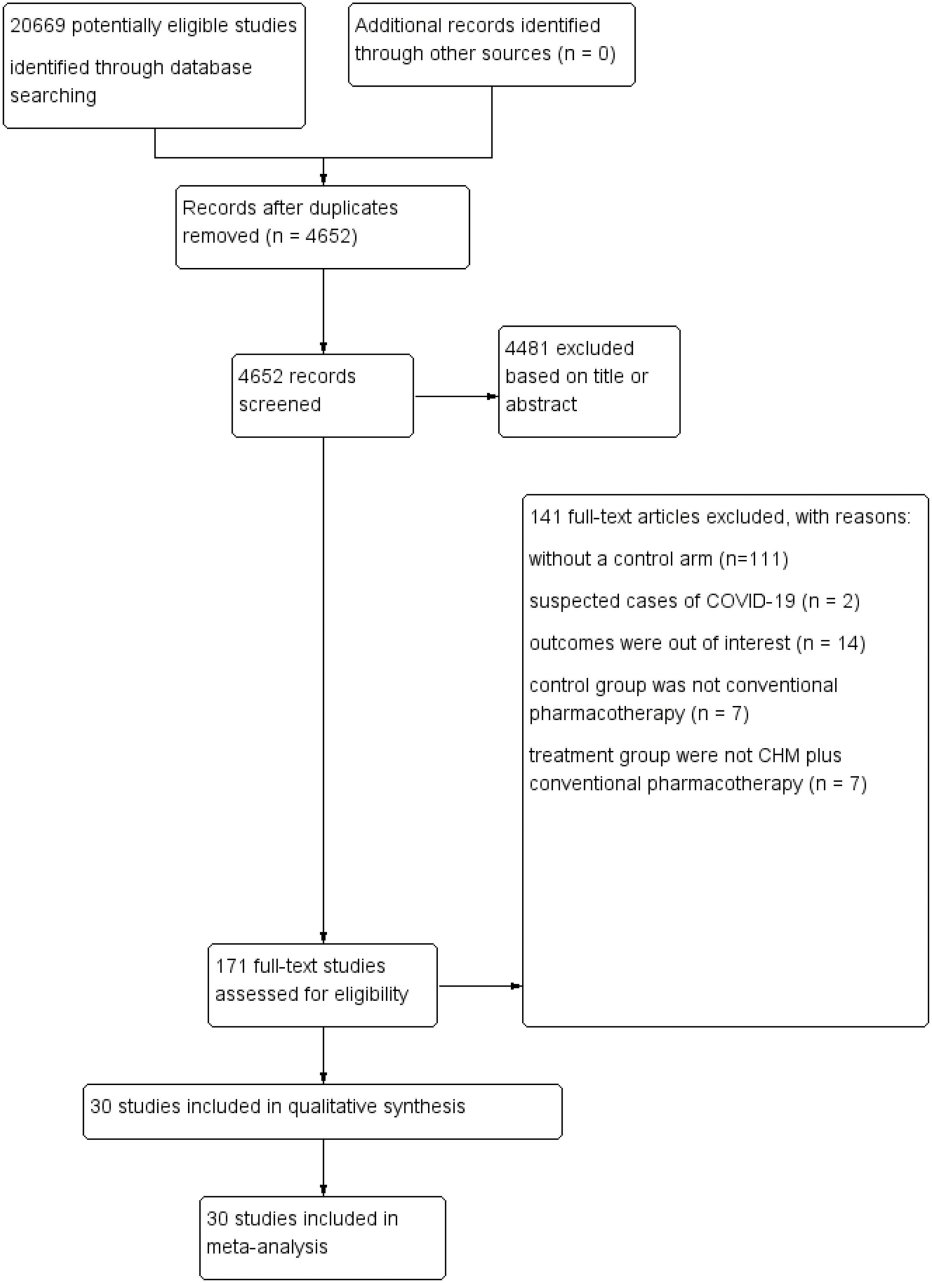
Flow diagram of search and selection process.

Detailed flow of the included studies was presented in Figure 1. In brief, systematic electronic searches yielded 20,669 potential citations for review initially. Then, 4, 652 unique records were remained after duplicate data abstraction. We removed 4,481 articles after cautious screening of titles and abstracts. Finally, of the 171 potential articles, 141 studies were excluded with reasons, and details were described in Figure 1. Finally, 30 studies that encompassed 3,145 COVID-19 cases were included and retrieved for quantitative synthesis in the current meta-analysis, after assessing the full-texts for eligibility.

### Description of Eligible Studies

There were 16 RCTs incorporating 1,226 patients with COVID-19 and 14 observational studies involving 1,919 patients with COVID-19 that met the inclusion criteria. All studies were performed in China, mainly in Wuhan, Hubei Province. The admission time was from December, 2019 to April, 2020. Lymphocyte count was reported in 6 RCTs (685 patients). CD4^+^ was included in 6 RCTs with 363 participants. CD8^+^ was reported in 4 RCTs with 324 patients, and CD4^+^/CD8^+^ ratio was reported in 3 RCTs with 244 patients. CD3^+^ was studied in 4 trials with330 participants. Leukocyte count was reported in 6 RCTs comprising of 590 patients, TNF-α was reported in 3 RCTs comprising of 243 patients, and IL-6 was reported in 6 researches comprising of 715 patients. All-cause mortality was reported in 9 studies (982 patients). Other details were shown in Table 2. The herbs that were used in the eligible studies have been showed in the Supplementary Table 1. Since the patients' comorbidities may interfere with the effects of CHM and alter their effects on the immune response, the comorbidities of the patients were also illustrated in the Supplementary Table 1.

**Table 2:** Basic characteristics of the observational studies.

	**Admission**	**Admission**	**Patients (n)**		**Age (year)**		**Gender (male %)**		**Baseline** **Leukocyte count** **(×10**^**9**^ **/L)**		**Lymphocyte** **count (×10**^**9**^ **/L)**		**Outcome**
	**Location**	**Time**	**I**	**C**	**I**	**C**	**I**	**C**	**I**	**C**	**I**	**C**	
Ai et al. (15)^a^	Guangzhou	Jan. 23, 2020–	33	34	52.33 ± 14.90	49.56 ± 16.30	63.60	55.90	NA	NA	NA	NA	➁
	Province	Mar. 17, 2020											➂
													➃
													➄
Ai et al. (24)^a^	Guangzhou	Jan. 23, 2020–	55	43	43.98 ± 12.60	45.95 ± 18.30	43.60	39.50	4.68 ± 1.40	4.83 ± 1.50	1.53 ± 0.60	1.50 ± 0.60	➀
		Mar. 3, 2020											
Chen et al. (25)^a^	Shenzhen,	Dec. 2019–	30	30	50.16 ± 5.11	49.52 ± 5.06	56.70	60.00	NA	NA	NA	NA	➉
	Guangzhou	Feb. 2020											⑪
	Province												⑬
Ding et al. (26)^a^	Wuhan,	Jan. 2020	51	49	54.70 ± 21.30	50.80 ± 23.50	76.50	79.60	NA	NA	NA	NA	➆
	Hubei Province												➇
Fu et al. (16)^a^	Guangzhou	Jan. 20, 2020–	37	36	45.26 ± 7.25	44.68 ± 7.45	51.40	52.80	5.07 ± 0.44	5.15 ± 0.36	1.52 ± 0.20	1.47 ± 0.22	➀
		Feb. 23, 2020											➁
													➂
													➃
													➅
Lan et al. (27)^a^	Shiyan,	Feb. 1, 2020–	43	42	43.05 ± 13.26	42.40 ± 14.47	79.10	71.40	4.61 ± 2.00	5.41 ± 1.15	1.68 ± 0.44	1.39 ± 0.77	➀
	Hubei	Feb. 20, 2020											➅
	Province												➇
Li and Zhang (28)^a^	Shanxi Province	Feb. 2020– Mar. 2020	6	6	52.00 ± 6.56	50.00 ± 10.00	66.70	50.00	2.50 ± 2.05	2.56 ± 1.87	NA	NA	➅
Ping et al. (29)^a^	Jiujiang, Jiangxi Province	Jan. 2020– Mar.2020	30	24	23-58	25-64	53.30	41.70	NA	NA	1.20 ± 0.36	1.15 ± 0.35	➀
Qiu et al. (30)^a^	Chongqing	Feb. 7, 2020–	25	25	53.35 ± 18.35	51.32 ± 14.62	52.00	56.00	NA	NA	NA	NA	➉
		Feb. 17, 2020											⑪
Wang et al. (31)^a^	Honghu,	Feb. 13, 2020–	40	40	41.10 ± 14.50	40.80 ± 13.70	57.50	70.00	3.43 ± 1.48	3.03 ± 1.51	0.42 ± 0.14	0.39 ± 0.21	➀
	Hubei Province	Mar. 18, 2020											➁
													➂
													➅
													➉
													⑪
													⑬
Wang et al. (32)^a^	Shijiazhuang, Hebei Province	Jan. 21,2020– Apr. 12, 2020	11	11	43.43 ± 17.51	41.73 ± 15.16	54.50	45.50	NA	NA	NA	NA	➉
Ye and Group (33)^a^	Wuhan, Hubei Province	Jan. 31, 2020– Feb. 19, 2020	28	14	65 (53.50–69)	59 (47–67)	7.40	28.60	NA	NA	NA	NA	➈
Yu et al. (34)^a^	Wuhan,	Feb. 17, 2020–	147	148	48.27 ± 9.56	47.25 ± 8.67	55.80	60.10	5.12 ± 0. 44	5.17 ± 0. 39	1.49 ± 0. 13	1.51 ± 0. 14	➀
	Hubei Province	Mar. 6, 2020											➅
													➈
Zhang et al. (35)^a^	Wuhan,	Jan. 31, 2020–	22	23	53.70 ± 3.50	55.60 ± 4.20	40.90	43.50	4.52 ± 1.98	4.68 ± 2.23	NA	NA	➅
	Hubei Province	Mar.3, 2020											➉
													⑪
													⑫
													⑬
Zhao et al. (36)^a^	Hefei,	Jan. 20, 2020–	24	15	NA	NA	53.30	58.30	4.57 (3.63,6.52)	4.22 (3.90,5.36)	NA	NA	➁
	Anhui Province	Feb. 24, 2020											➆
Zhou et al. (17)^a^	Changsha	Before Feb. 22, 2020	52	52	52.47 ± 10.99	51.11 ± 9.87	61.50	53.80	NA	NA	NA	NA	➁
													➂
													➃
													➄
													➆
Chen et al. (37)^b^	Wuhan, Hubei Province	Before Mar. 20, 2020	156	156	57 (45, 68)	66 (54, 76)	41.70	52.80	5.46 (4.42, 7.00)	5.02 (3.83, 6.60)	1.44 (1.01, 1.88)	1.19 (0.84, 1.58)	➈
Chen et al. (38)^b^	Wuhan, Hubei Province	Jan. 20, 2020– Feb. 20, 2020	100	100	60.20 ± 6.60	60.40 ± 6.60	66.00	64.00	2.70 ± 0.30	2.60 ± 0.20	NA	NA	➇
Chen et al. (39)^b^	Wuhan, Hubei Province	Jan. 25, 2020– Mar. 18, 2020	115	115	63.02 ± 13.61	60.17 ± 16.02	47.80	40.90	NA	NA	NA	NA	⑫
Hu et al. (40)^b^	Guangxi Province	Jan. 30, 2020– Mar. 5, 2020	31	21	48.30 ± 16.56	49.75 ± 17.15	64.50	66.70	6.08 ± 2.75	5.67 ± 2.43	NA	NA	➈
Huang et al. (41)^b^	Wuhan,	Feb. 11, 2020–	30	15	58.40 ± 15. 50	66.30 ± 14. 10	43.30	60.00	NA	NA	NA	NA	➈
	Hubei Province	Mar. 12, 2020											⑫
Ke et al. (42)^b^	Wuhan, Hubei Province	Jan. 2020– Mar. 2020	81	22	56.17 ± 13.35	52.43 ± 10.12	56.80	59.10	4.63 ± 1.81	4.26 ± 1.75	1.04 ± 0.42	0.93 ± 0.37	⑫
Qian et al. (43)^b^	Wuhan, Hubei Province	Feb. 2020– Apr. 2020	170	130	41.02 ± 5.36	42.63 ± 5.89	54.10	53.80	5.77 ± 1.87	5.73 ± 1.57	1.33 ± 0.56	1.31 ± 0.57	⑫
Yu et al. (44)^b^	Suizhou, Hubei Province	Jan. 29, 2020– Mar. 15, 2020	75	75	17-86	17-86	NA	NA	NA	NA	NA	NA	➇
Wang et al. (45)^b^	Wuhan, Hubei Province	Early stage of COVID-19 outbreak	47	40	44.68 ± 11.42	49.70 ± 13.13	40.40	47.50	NA	NA	NA	NA	➈
Wang et al. (32)^b^	Wuhan, Hubei Province	Jan. 15, 2020– Mar. 30, 2020	43	43	NA	NA	NA	NA	NA	NA	NA	NA	➈
Xin et al. (46)^b^	Xiangyang, Hubei Province	Jan. 24, 2020– Feb. 15, 2020	37	26	46.10 (23.50–89.90)	50.70 (15.30–81.90)	46.00	46.20	4.82 (3.67–5.52)	4.29 (3.39–5.08)	NA	NA	➈
Yu et al. (34)^b^	Wuhan, Hubei Province	Feb. 10, 2020– Apr. 1, 2020	43	46	64.23 ± 2.51	60.50 ± 2.08	48.80	39.10	NA	NA	NA	NA	➄
													➇
Zhang et al. (35)^b^	Chongqing	Feb. 1, 2020– Mar. 5, 2020	90	30	51.70 ± 12.50	49.20 ± 13.60	51.10	53.30	NA	NA	NA	NA	➇
Zhang et al. (47)^b^	Shanghai	Jan. 26, 2020– Apr. 15, 2020	25	57	33 (23–53)	38 (29–58)	44.00	40.35	NA	NA	NA	NA	➄

*C, control group; I, intervention group; NA, not applicable*.

*^a^RCT; ^b^observational study; ➀ Lymphocyte count; ➁ CD4^+^; ➂ CD8^+^; ➃ CD4^+^/CD8^+^ ratio; ➄ CD3^+^; ➅ Leukocyte count; ➆ TNF-α; ➇ IL-6; ➈ All-cause mortality; ➉ Time to the remission of fever; ⑪ Time to cough remission; ⑫ Time to the remission of chest tightness; ⑬ time to fatigue remission*.

### Assessment of Methodological Quality

Considering randomization, nine RCTs described the methodology of random-sequence generation (15, 17, 24–26, 30, 31, 34, 48). The blinding of subjects and researcher was reported in one study (33), and the other RCTs did not reported the methodology of blinding. Participant withdrawal was low to zero in most studies. Six studies (16, 27–29, 35, 36) were considered as being of low quality (modified Jadad score <4). The score of ten studies (15, 17, 24–26, 30, 31, 33, 34, 48) obtained a modified Jadad score of 4 and they were judged to be of high quality. In terms of observational studies, the NOS scores were 5–8, indicating most of the studies were of low risk of bias. The detailed results of the quality assessment of the included studies were presented in Tables 3, 4.

**Table 3:** Modified Jadad scale for the included RCTs.

	**Generation of randomization**	**Randomization allocation**	**Blinding**	**Dropouts and withdrawals**	**Modified**	Quality^*^
	**Allocation sequence (0–2 points)**	**Concealment (0–2 points)**	**(0–2 points)**	**(0–1 point)**	**Jadad scale**	
Ai et al. (15)	2	1	0	1	4	High
Ai et al. (24)	2	1	0	1	4	High
Chen et al. (25)	2	1	0	1	4	High
Ding et al. (26)	2	1	0	1	4	High
Fu et al. (16)	1	0	0	1	2	Low
Lan et al. (27)	0	0	0	1	1	Low
Li and Zhang (28)	1	0	0	1	2	Low
Ping et al. (29)	0	0	0	1	1	Low
Qiu et al. (30)	2	1	0	1	4	High
Wang et al. (31)	2	1	0	1	4	High
Wang et al. (32)	2	1	0	1	4	High
Ye and Group (33)	1	1	1	1	4	High
Yu et al. (34)	2	1	0	1	4	High
Zhang et al. (35)	1	0	0	1	2	Low
Zhao et al. (36)	1	0	0	0	1	Low
Zhou et al. (17)	2	1	0	1	4	High

* *We considered ≤ 2 score as low, 3 score as moderate and ≥4 score as high quality*.

**Table 4:** Newcastle-Ottawa risk of bias assessment.

	**Selection**				**Comparability**		**Outcome**			**Score**	**Risk of bias**
	**1**	**2**	**3**	**4**	**5**	**6**		**7**	**8**		
	**The exposed**	**The non-exposed**	**Ascertainment of exposure**	**Outcome was not present at start of study**	**Age**	**other factor**	**Assessment of outcome**	**Was follow-up long enough for outcome to occur**	**Complete accounting**		
Chen et al. (37)	1	1	1	1	1	0	1	1	0	7	L
Chen et al. (38)	1	1	1	0	1	1	1	1	0	7	L
Chen et al. (39)	1	1	1	0	1	1	1	1	0	7	L
Hu et al. (40)	1	1	1	0	0	0	1	1	0	5	M
Huang et al. (41)	1	1	1	1	1	1	1	1	0	8	L
Ke et al. (42)	1	1	1	1	1	1	1	0	0	7	L
Qian et al. (43)	1	1	1	0	1	1	1	1	0	7	L
Yu et al. (44)	1	1	1	0	0	0	1	1	0	5	M
Wang et al. (31)	1	1	1	1	1	1	1	0	0	7	L
Wang et al. (32)	1	1	1	1	1	1	1	1	0	8	L
Xin et al. (46)	1	1	1	1	1	1	1	1	0	8	L
Yu et al. (34)	1	1	1	0	1	1	1	1	0	7	L
Zhang et al. (35)	1	1	1	1	1	1	1	1	0	8	L
Zhang et al. (47)	1	1	1	1	1	1	1	1	0	8	L

### Outcomes of CHM Plus Routine Treatment vs. Routine Treatment

#### Lymphocyte Count

**Figure 2:**
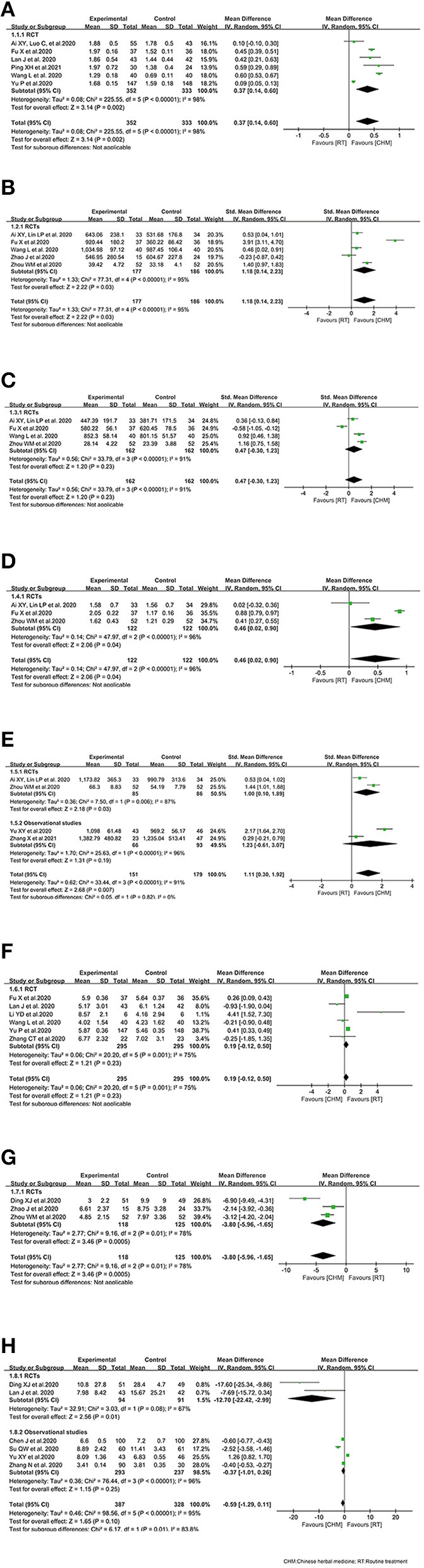
Effect of Chinese herbal medicine as an adjuvant on immunity profiles during COVID-19. **(A)** Lymphocyte count. **(B)** CD4^+^. **(C)** CD8^+^. **(D)** CD4^+^/CD8^+^. **(E)** CD3^+^. **(F)** Leukocyte count. **(G)** TNF-α. **(H)** IL-6.

Pooled estimates from 6 RCTs (16, 24, 27, 29, 31, 34) showed improvement effect on lymphocyte count in CHM arm. The combined WMD of lymphocyte count showed significant increase on lymphocyte count in CHM arm [WMD 0.37 (95% CI 0.14–0.60); *P* = 0.002; *I*^2^ = 98 %]. Random effect model was used because of the considerable heterogeneity (Figure 2A).

#### CD4^+^

Five RCTs (15–17, 31, 36) reported CD4^+^ in COVID-19 patients treated with CHM. The combined SMD of CD4^+^ was 1.18 (95% CI, 0.14–2.23, *P* = 0.03). CHM plus routine treatment showed a superior ability for CD4^+^ improvement. We used random effect model since significant heterogeneity was observed among the studies (*I*^2^ = 95%, *P* < 0.00001) (Figure 2B).

#### CD8^+^

Four RCTs (15–17, 31) reported the effect of CHM on CD8^+^ during COVID-19. The pooled analysis showed inconclusive effects on CD8^+^ between the CHM and control treatment groups among COVID-19 patients, of which, the combined SMD of CD8^+^ was 0.47 (95% CI, −0.30–1.23, *P* = 0.23; random effect model) (Figure 2C).

#### CD4^+^/CD8^+^ Ratio

Three RCTs (15–17) reported the effect of CHM on CD4^+^/CD8^+^ ratio in COVID-19 patients. A superior ability for CD4^+^/CD8^+^ improvement was observed in RCTs [WMD = 0.46, *P* = 0.04; 95% CI (0.02, 0.90); random effect model] (Figure 2D).

#### CD3^+^

Only two RCT (15, 17) reported the effect of CHM as an adjuvant on CD3^+^ during COVID-19, and observational studies (34, 47) were included considering the limited number of RCTs. The combined SMD of CD8^+^ was 1.11 (95% CI, 0.30–1.92, *P* = 0.007; random effect model), and CHM plus routine treatment showed a superior ability for CD3^+^ improvement, compared with routine treatment alone. Similar result was identified in the RCT subgroup [1.00 (0.10, 1.89)] (Figure 2E).

#### Leukocyte Count

The overall analysis of leukocyte count included 6 RCTs (16, 27, 28, 31, 34, 35). Together, 295 COVID-19 cases with CHM exposure and 295 COVID-19 cases with control treatment were included. There was no significant difference between the CHM arm and the control arm in terms of leukocyte count in RCTs (WMD = 0.19, 95% CI, −0.12, 0.50, *P* = 0.23) (Figure 2F).

#### TNF-α

Three RCTs (17, 26, 36) reported the effect of oral CHM as an adjuvant on TNF-α during COVID-19. The pooled analysis showed decreased effects on TNF-α between the CHM and control treatment groups among COVID-19 patients, of which, the combined WMD of TNF-α was −3.80 (95% CI, −5.96, −1.65, *P* = 0.0005; random effect model) (Figure 2G).

#### IL-6

Only two RCTs (26, 27) reported the effect of oral CHM as an adjuvant on IL-6 during COVID-19. Thus, observational studies (34, 35, 38, 44) were also considered when the meta-analysis was conducted. A possible but uncertain decrease of IL-6 in CHM arm was detected in the overall effect (WMD = −0.59, 95% CI, −1.29, 0.11, *P* = 0.10). While, a decrease of IL-6 in CHM arm was detected in the subgroup of RCTs (Figure 2H).

#### All-Cause Mortality

**Figure 3:**
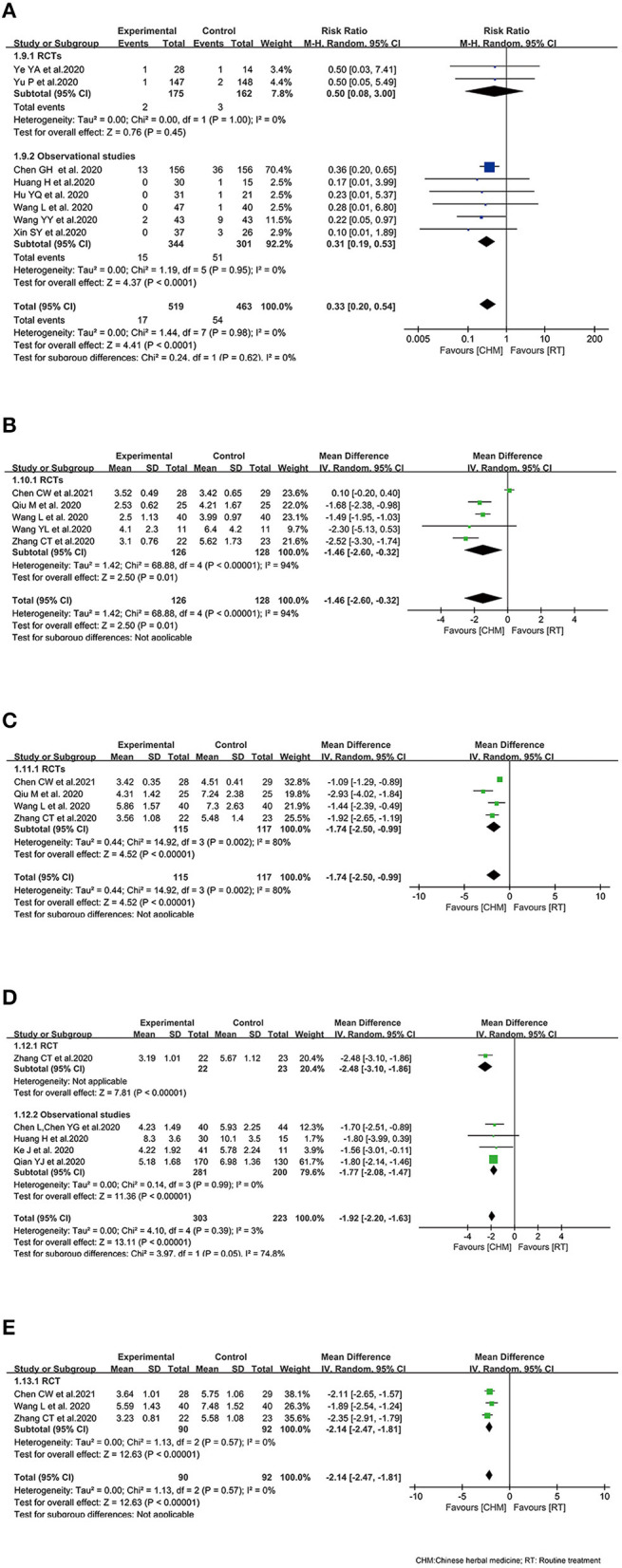
Effect of Chinese herbal medicine as an adjuvant on clinical profiles during COVID-19. **(A)** All-cause mortality. **(B)** Time to the remission of fever. **(C)** Time to cough remission. **(D)** Time to the remission of chest tightness. **(E)** Time to fatigue remission.

Only two RCTs (33, 34) investigated all-cause mortality of COVID-19 patients treated with oral CHM, and observational studies (32, 37, 40, 41, 45, 46) were included due to the inadequate number of RCTs. The outcome of all-cause mortality was pooled with 519 COVID-19 patients in CHM arm and 463 COVID-19 patients in control arm among the eligible 8 studies. The pooled result showed that the mortality of interventions was lower than that of comparator group (RR 0.33, 95% CI 0.20–0.54, *I*^2^ = 0%). In subgroup analysis of study design, similar results were observed in observational studies between the treatment arm and the control arm (RR 0.31, 95% CI 0.19–0.53, *I*^2^ = 0%) (Figure 3A).

#### Time to the Remission of Fever

Five RCTs recruiting 254 patients (25, 30, 31, 35, 48) reported the time to the remission of fever. It was documented that CHM plus control treatment could reduce the time to fever remission (WMD −1.46, 95% CI −2.60, −0.32). Random effect model was used because of the heterogeneity among the studies (*I*^2^ = 94%, *P* < 0.00001) (Figure 3B).

#### Time to Cough Remission

Regarding the time to cough remission, four RCTs (25, 30, 31, 35) including 232 patients documented data on it. The combination of CHM and control treatment appeared to show a superiority in reducing the time to cough remission with WMD of −1.74 (95% CI −2.50, −0.99) (Figure 3C).

#### Time to the Remission of Chest Tightness

In terms of the time to the remission of chest tightness, 5 studies reported time to the remission of chest tightness (35, 39, 41–43). The integrated data demonstrated that the time to the remission of chest tightness in intervention arm was shorter than that in control arm (WMD −1.92, 95% CI −2.20, −1.63; *I*^2^ = 3%). The subgroup analysis based on study design showed similar results (Figure 3D).

#### Time to Fatigue Remission

For the time to fatigue remission, there was three RCTs (25, 31, 35) included, indicating that patients received oral CHM as an adjuvant medicine had shorter fatigue duration than patients who received control treatment alone (WMD −2.14, 95% CI −2.47, −1.81; *I*^2^ = 0%) (Figure 3E).

#### Side Effects and Complications

The common side effects like nausea (25), diarrhea (25, 31, 47), liver injury (17), and increased D-dimer (32), were studied in 12 included trials (17, 25–29, 31, 32, 38–40, 47). One RCT reported the frequency of complications, founding that there was no difference between the two groups, indicating that CHM did not increase complications (33).

**Figure 4:**
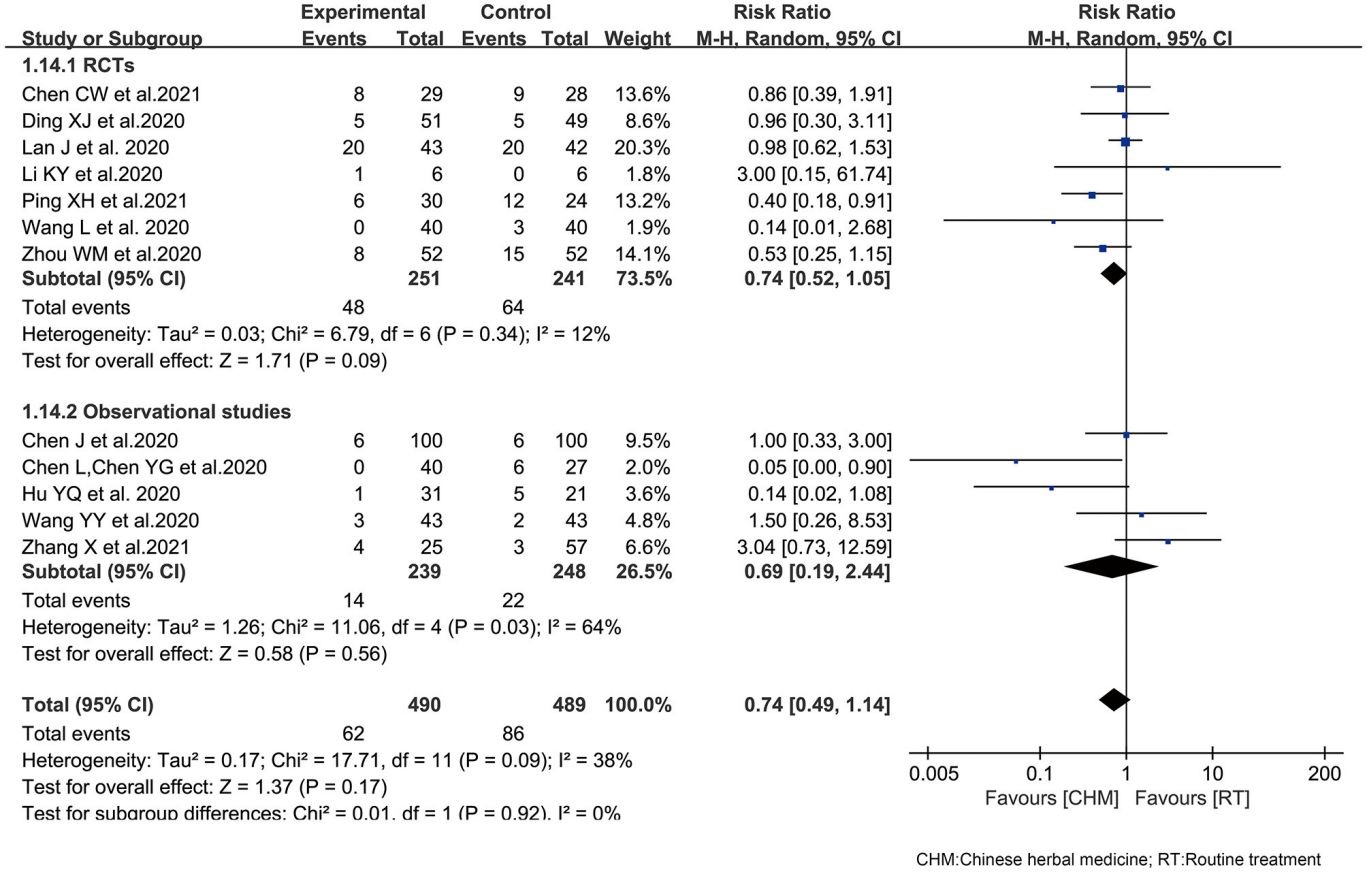
The effect of Chinese herbal medicine as an adjuvant on side effects during COVID-19.

The side effects of CHM were assessed by meta-analysis, and there was no significant difference between the treatment arm and the control arm. The result demonstrated that oral CHM plus routine treatment did not increase side effects compared with routine treatment used alone, and even accompanied with a trend of decreasing side effects (RR 0.74, 95% CI 0.49, 1.14; *I*^2^ = 38%). Although one of the main concerns on using herbal medicine was their interaction with other medications and side effects, the clinical trials available showed that CHM combination therapy did not contribute to more side effects, and was relatively safe. Subgroups of RCTs and observational studies showed the similar results (Figure 4).

### Sensitivity Analysis and Publication Bias

**Figure 5:**
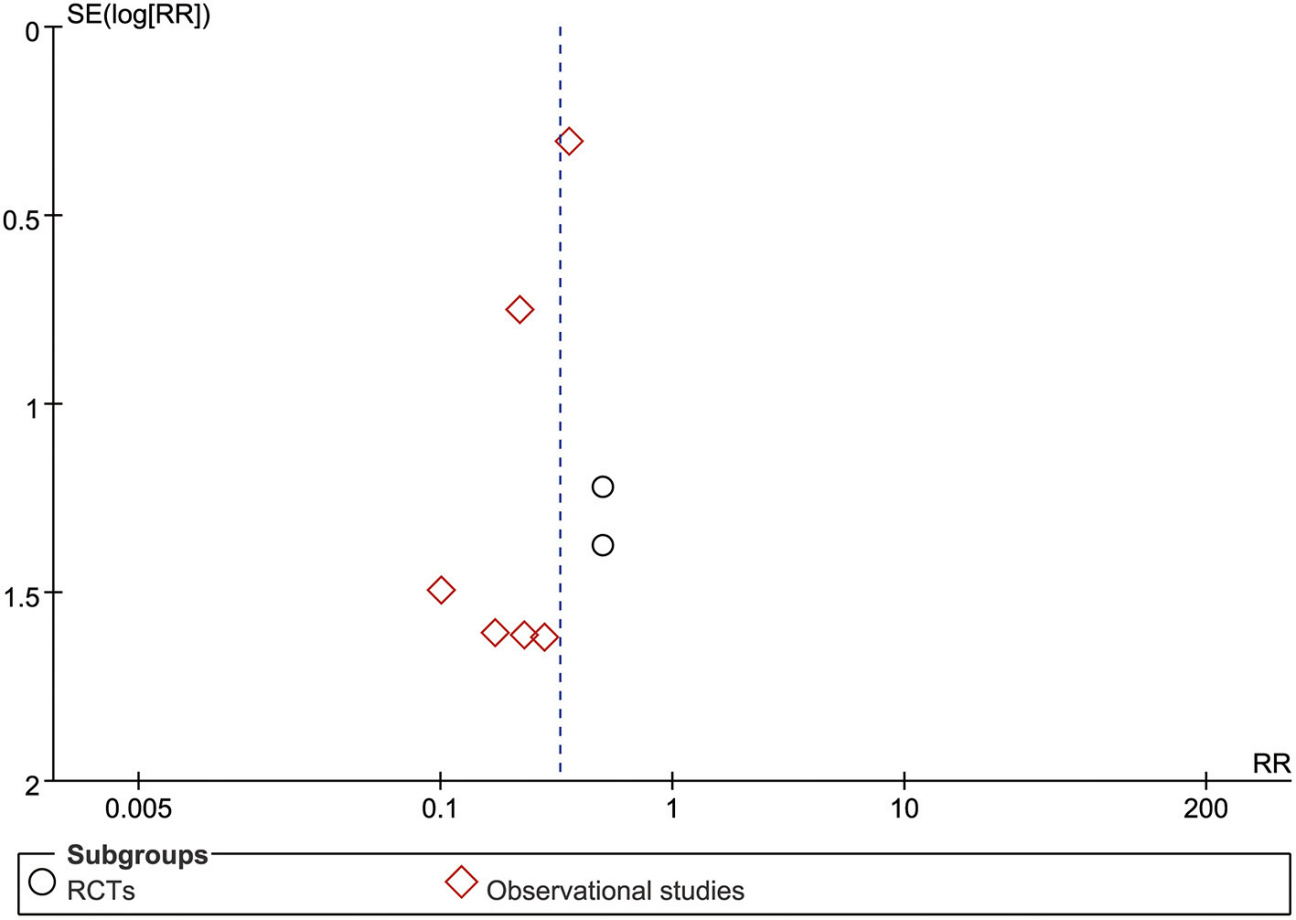
Funnel plot.

Sensitivity analysis revealed that the overall estimates of lymphocyte counts, all-cause mortality, side effects, and the remission time of cough, chest tightness, and fatigue were not influenced by elimination of any study, indicating that these results were credible. Nevertheless, sensitivity analysis suggested that the results of CD4^+^, CD8^+^, CD4^+^/CD8^+^ ratio, CD3^+^, the remission time of fever, and leukocyte count were not robust based on the inclusive studies currently. An asymmetry in the meta-analysis of all-cause mortality (Figure 5) was noted by the visual inspection of funnel plot, indicating potential publication bias.

### Certainty of the Evidence

The certainty of evidence was assessed using the GRADEpro software, and the results were outlined in Table 5. The outcome quality of all-cause mortality in RCTs was high. The evidence with moderate quality included all-cause mortality in observational studies; the time to the remission of fever, cough, fatigue, and chest tightness in RCTs; CD8^+^, CD4^+^/CD8^+^, CD3^+^, and side effects in RCTs. The outcomes of lymphocyte count, CD4^+^, leukocyte count, TNF-α, and IL-6 in RCTs had low quality of evidence. Additionally, the quality of findings relevant to CD3^+^, IL-6, the time to the remission of chest tightness, and side effects in observational studies was very low.

**Table 5:** Certainty of evidence.

**Quality assessment**							**No of patients**		**Effect**		**Quality**	**Importance**
**No. of studies**	**Design**	**Risk of bias**	**Inconsistency**	**Indirectness**	**Imprecision**	**Other considerations**	**CHM group**	**Control group**	**Relative (95% CI)**	**Absolute**		
**Lymphocyte count—RCT (better indicated by lower values)**												
6	Randomized trials	Serious^a^	Serious^b^	No serious indirectness	No serious imprecision	None	352	333	–	MD 0.37 higher (0.14–0.6 higher)	⊕⊕○○ Low	Critical
**CD4** ^**+**^ **–RCTs (better indicated by lower values)**												
5	Randomized trials	Serious^a^	Serious^b^	No serious indirectness	No serious imprecision	None	177	186	–	SMD 1.18 higher (0.14–2.23 higher)	⊕⊕○○ Low	Critical
**CD8** ^**+**^ **–RCTs (better indicated by lower values)**												
4	Randomized trials	No serious risk of bias	Serious^b^	No serious indirectness	No serious imprecision	None	162	162	–	SMD 0.47 higher (0.3 lower to 1.23 higher)	⊕⊕⊕○ Moderate	Critical
**CD4** ^**+**^ **/CD8** ^**+**^ **—RCTs (better indicated by lower values)**												
3	Randomized trials	No serious risk of bias	Serious^b^	No serious indirectness	No serious imprecision	None	122	122	–	MD 0.46 higher (0.02–0.9 higher)	⊕⊕⊕○ Moderate	Critical
**CD3** ^**+**^ **–RCTs (better indicated by lower values)**												
2	Randomized trials	No serious risk of bias	Serious^b^	No serious indirectness	No serious imprecision	None	85	86	–	SMD 1 higher (0.1–1.89 higher)	⊕⊕⊕○ Moderate	Critical
**CD3** ^**+**^ **–observational studies (better indicated by lower values)**												
2	Observational studies	No serious risk of bias	Serious^b^	No serious indirectness	No serious imprecision	None	66	93	–	SMD 1.23 higher (0.61 lower to 3.07 higher)	⊕○○○ Very low	Critical
**Leukocyte count—RCT (better indicated by lower values)**												
6	Randomized trials	Serious^a^	Serious^b^	No serious indirectness	No serious imprecision	None	295	295	–	MD 0.19 higher (0.12 lower to 0.5 higher)	⊕⊕○○ Low	Important
**TNF-α–RCTs (better indicated by lower values)**												
3	Randomized trials	Serious^a^	Serious^b^	No serious indirectness	No serious imprecision	None	118	125	–	MD 3.8 lower (5.96–1.65 lower)	⊕⊕○○ Low	Important
**IL-6—RCTs (better indicated by lower values)**												
2	Randomized trials	Serious^a^	Serious^a^	No serious indirectness	No serious imprecision	None	94	91	–	MD 12.7 lower (22.42–2.99 lower)	⊕⊕○○ Low	Important
**IL-6—Observational studies (better indicated by lower values)**												
4	Observational studies	No serious risk of bias	Serious^b^	No serious indirectness	No serious imprecision	None	293	237	–	MD 0.37 lower (1.01 lower to 0.26 higher)	⊕○○○ Very Low	Important
**Mortality—RCTs**												
2	Randomized trials	No serious risk of bias	No serious inconsistency	No serious indirectness	No serious imprecision	None	2/175 (1.1%)	3/162 (1.9%)	RR 0.5 (0.08–3)	9 fewer per 1,000 (from 17 fewer to 37 more)	⊕⊕⊕⊕ High	Critical
								4.3%		22 fewer per 1,000 (from 40 fewer to 86 more)		
**Mortality—Observational studies**												
6	Observational studies	No serious risk of bias	No serious inconsistency	No serious indirectness	No serious imprecision	Strong association^c^	15/344 (4.4%)	51/301 (16.9%)	RR 0.31 (0.19–0.53)	117 fewer per 1,000 (from 80 fewer to 137 fewer)	⊕⊕⊕○ Moderate	Critical
								9.1%		63 fewer per 1,000 (from 43 fewer to 74 fewer)		
**Time to remission of fever—RCTs (better indicated by lower values)**												
5	Randomized trials	No serious risk of bias	Serious^b^	No serious indirectness	No serious imprecision	None	126	128	–	MD 1.46 lower (2.6–0.32 lower)	⊕⊕⊕○ Moderate	Important
**Time to cough remission—RCTs (better indicated by lower values)**												
4	Randomized trials	No serious risk of bias	Serious^b^	No serious indirectness	No serious imprecision	None	115	117	–	MD 1.74 lower (2.5–0.99 lower)	⊕⊕⊕○ Moderate	Important
**Time to the remission of chest tightness—RCT (better indicated by lower values)**												
1	Randomized trials	Serious^a^	No serious inconsistency	No serious indirectness	No serious imprecision	None	22	23	–	MD 2.48 lower (3.1–1.86 lower)	⊕⊕⊕○ Moderate	Important
**Time to the remission of chest tightness—Observational studies (better indicated by lower values)**												
4	Observational studies	Serious^e^	No serious inconsistency	No serious indirectness	No serious imprecision	None	281	200	–	MD 1.77 lower (2.08–1.47 lower)	⊕○○○ VERY low	Important
**Time to fatigue remission—RCT (better indicated by lower values)**												
3	Randomized trials	Serious^a^	No serious inconsistency	No serious indirectness	No serious imprecision	None	90	92	–	MD 2.14 lower (2.47–1.81 lower)	⊕⊕⊕○ MODERATE	Important
**Side effects—RCTs**												
7	Randomized trials	Serious^a^	No serious inconsistency	No serious indirectness	No serious imprecision	None	48/251 (19.1%)	64/241 (26.6%)	RR 0.74 (0.52–1.05)	69 fewer per 1,000 (from 127 fewer to 13 more)	⊕⊕⊕○ Moderate	Important
								28.9%		75 fewer per 1,000 (from 139 fewer to 14 more)		
**Side effects—observational studies**												
5	Observational studies	No serious risk of bias	Serious^b^	No serious indirectness	No serious imprecision	None	14/239 (5.9%)	22/248 (8.9%)	RR 0.69 (0.19–2.44)	28 fewer per 1,000 (from 72 fewer to 128 more)	⊕○○○ Very low	Important
								6%		19 fewer per 1,000 (from 49 fewer to 86 more)		

a *The average Jadad score ≤ 3 because the generation of randomization allocation sequence, randomization allocation, or the blinding are unclear, and we decided to downgrade the quality of evidence as risk of bias*.

b *There is serious heterogeneity among the studies included in the analysis of this outcome. Overall, we decided to downgrade by one level when considering these issues along with inconsistency*.

*^c^RR < 0.5 based on consistent evidence from at least two studies*.

*^d^Visual inspection of the funnel plot showed a potential asymmetry in the meta-analysis*.

*^e^The average NOS score of this outcome ≤ 6. Therefore, we decided to downgrade the quality of evidence*.

## Discussion

### Interpretation of the Outcomes

Taken together, we uncovered the key role of oral CHM in COVID-19 treatment through 30 studies involving 3,145 COVID-19 patients in the present study. Compared to those in control arms, patients received oral CHM as an adjuvant have significantly better lymphocyte counts, CD3^+^, CD4^+^, and CD4^+^/CD8^+^ ratio. CHM interventions notably reduced TNF-α and all-cause mortality, and shortened the time to symptoms remission, including the remission of fever, cough, fatigue, and chest tightness, without significant effect on leukocyte count, CD8^+^, and IL-6, based on the present studies.

Compared with routine treatment based on western medicine, CHM, improving lymphocyte counts, might improve host antiviral immune response, since protective and enduring immune responses to COVID-19 usually arose from the actions of lymphocytes (49). Lymphocytopenia was common in COVID-19 patients, especial in patients with severe disease (50). CHM may be recommended to patients with severe COVID-19 in clinical practice, especially those accompany with lymphocytopenia. Besides, T cells were associated with the effective immune response to SARS-CoV-2 (3). Since CHM had positive effect on CD3 T-lymphocyte, CD4 T-lymphocyte, and CD4^+^/CD8^+^ ratio, and reduced TNF-α and mortality, the survival rate may be increased by CHM resulting from the potential immunopharmaceutical effects. The effect of CHM on improving clinical parameters might be related to the process of CHM modulating immune effector cells during COVID-19.

The combination of CHM and routine treatment was supposed to show improved CD8^+^ and reduced IL-6. Nonetheless, limited evidence was found to support this idea in the present studies. No convincing evidence that CHM interventions improved CD8^+^ and reduced IL-6 was found based on currently available studies. The meta-analysis illuminated inconsistent results for IL-6: observational studies showed that there was no significant difference between CHM and routine treatment, while RCTs signified noteworthy reduced IL-6 in CHM group compared with control arm. The authors speculated the reason underlying the inconsistent IL-6 results was the small sample size of these included studies available for meta-analysis.

Abnormal leukocyte count was described as a potential indicator of the severity of respiratory symptoms and a poor clinical outcome in COVID-19 recently, but the exact mechanism was not clear (50). Available evidence evaluating the effect of CHM used in COVID-19 was not solid enough to draw definite conclusion concerning how leukocyte count changed. The effect of CHM on leukocyte count was not clear in the present meta-analysis, though the most common mechanisms and targets aimed by CHM based on these studies available up to now, have been not only antiviral, but anti-inflammatory, which probably by alleviating the “cytokine storm.” For instance, an *in vitro* study suggested that Lianhuaqingwen significantly inhibited SARS-CoV-2 replication, affected virus morphology, and exerted anti-inflammatory activity, markedly reducing TNF-α, IL-6, CCL-2/MCP-1 and CXCL-10/IP-10 production (51). The aims of the plant studies in general were to play anti- antiviral, inflammatory, antipyretic, antitussive, expectorant, antiasthmatic, and even immunological effects, possessing a wide range of pharmacological functions (52). Nonetheless, more researches on clinical evidence and molecular mechanism by CHM are warranted.

The ongoing pandemic of COVID-19 has led to 76,250,431 confirmed cases and 2,531,542 deaths globally, as of 2 March 2021 (53), causing overwhelming burden on health-care systems. Upon the emergency of SARS-CoV-2, reducing mortality and the time to clinical symptoms remission were of utmost urgency. Even if routine treatments are helpful in COVID-19, by the time they play effects, the pandemic's human and economic cost will have been enormous (54). There remains an unmet need for achieving symptom control quickly. It is of huge importance to provide a fast, cost-effective, and immediately available pharmaceutical solution to curb the global spread of SARS-CoV-2. CHM as an adjuvant, with the advantage of accelerating the recovery of clinical symptoms, might shorten the treatment duration, which may reduce the further impact of pandemic and the burden to healthcare facilities. Therefore, it would be better if CHM complement early control measures in clinical practice, which may be pivotal for combating COVID-19 pandemic.

Although Chinese traditional medicine products were employed to treat COVID-19 in clinical practice, and the immunomodulatory effects of anti-COVID-19 TCM formulae have been evaluated by multiple virus-related pathways (55), there was not sufficient supportive experimental evidence for the action of CHM against SARS-CoV-2, as well as the exact mechanism of action. The use of CHM in recent trials on COVID-19 patients requires further evaluation and investigation. As for CHM as an alternative medicine, the number of relevant studies available is limited. Up to date, we could not recommend CHM alone superior to the routine treatment of COVID-19, and larger RCTs in future clinical trials with sensitive endpoints are needed to verify the value of CHM as an alternative medicine for SARS-CoV-2 infection.

### Strengths and Limitations

First of all, the present detailed systematic review and meta-analysis exploring the latest clinical evidence for immunological profiles of COVID-19 patients treated with oral CHM as an adjuvant, would be beneficial for us to understand the lesser-known roles of CHM on lymphocyte, CD3^+^, CD4^+^, CD4^+^/CD8^+^, and TNF-α, which will deepen our insights into COVID-19 treatments. Secondly, our search strategy and eligibility criteria were comprehensive, and the present study objectively and rigorously assessed the immunological profiles and all-cause mortality of CHM therapies for COVID-19 patients, which might facilitate our understanding of the optimal treatment for COVID-19 patients, and help inform pandemic containment strategies. Thirdly, the certainty of the evidence was evaluated with the GRADE approach. Fourthly, the level of evidence for the present study was relatively high, compared the previous animal experiments, vitro cell tests, and data mining investigating the immunogenicity of CHM against SARS-CoV-2, because our study was based on clinical and real-world studies.

Admittedly, there are a few limitations that must be acknowledged. The data of some outcomes currently available is sparse, such as CD3^+^ and IL-6, causing imprecision of outcomes, and the certainty of evidence was downgraded. Besides, the development of an epidemic disease has different stages, so treatment is provided according to manifestations at a specific stage based on diagnosis and treatment of Chinese medicine. Therefore, this might be a factor contributing to the leukocyte count results presented in the present review as trials might not differentiate stages of the development of COVID-19 when herbs were consumed. Moreover, the inclusive RCTs with open-label design restricted the quality grade of effects. In addition, how the treatment was applied differed and reporting was limited. Additionally, the main drawback of this meta-analysis is the heterogeneity of included cases, because of the various CHM used in different studies. But the different CHMs were all aimed at reducing dampness, an important pathogenic factor in traditional Chinese medicine (TCM), and they have some homogeneity based on TCM theory. CHM containing multiple herbs is supposed to get assessed separately in the future research with the increasing number of RCTs studying specific preparations containing the same herbs. In general, those results should be interpreted with caution considering the small sample size of some studies and other risk of low quality.

## Conclusion

CHM as an adjuvant medicine may show improvement effects on lymphocyte counts, CD4^+^, CD4^+^/CD8^+^, and CD3^+^, and reduce TNF-α, the risk of all-cause mortality, the time to the remission of clinical manifestations including cough, fever, chest tightness, and fatigue, compared with the routine treatment alone. Our results may suggest clinicians using oral CHM as an adjuvant to achieve better immune improvement and symptom control in shorter time with a lower risk of all-cause mortality in response to the COVID-19 pandemic. CHM might improve the clinical parameters through the immunity profile, and further high-quality RCTs are warranted to confirm the results. Large RCTs objectively evaluating the efficacy of CHM on immune responses in COVID-19 are necessary to confirm our findings.

## Data Availability Statement

The original contributions presented in the study are included in the article/Supplementary Material, further inquiries can be directed to the corresponding author/s.

## Author Contributions

ZW and SS were responsible for the study design. FW provided administrative management. SS and HY performed the selection of literatures. Then, the data was evaluated and analyzed by SS, WL, SK, and YWu. Moreover, SS, XW, CP, and DH participated in writing manuscript. WL, BC, YWa, PZ, YH, and ZW revised the manuscript for important intellectual content. Finally, all authors approved the full manuscript.

## Funding

This work was supported by the China Postdoctoral Science Foundation (Grant No. 2020T130010ZX).

## Conflict of Interest

The authors declare that the research was conducted in the absence of any commercial or financial relationships that could be construed as a potential conflict of interest.

## Publisher's Note

All claims expressed in this article are solely those of the authors and do not necessarily represent those of their affiliated organizations, or those of the publisher, the editors and the reviewers. Any product that may be evaluated in this article, or claim that may be made by its manufacturer, is not guaranteed or endorsed by the publisher.
