# Supplementary material for: Oral Chinese Herbal Medicine on Immune Responses During Coronavirus Disease 2019: A Systematic Review and Meta-Analysis
Source: Front Med (Lausanne). 2022 Jan 21;8:685734. doi: 10.3389/fmed.2021.685734 (PMC8814103; doi:10.3389/fmed.2021.685734)
Supplement: Supplementary file 4 [file Data_Sheet_1.docx]

**Search strategies**

| Literature databases | Search items | Items found |
| --- | --- | --- |
| (1) PubMed | (("Medicine, Chinese Traditional"[MeSH Terms]) OR (Search: (((((((((((((((((((("Traditional Chinese Medicine") OR ("Traditional Medicine, Chinese")) OR ("Hsueh, Chung I")) OR ("Zhong Yi Xue")) OR ("Chinese Traditional Medicine")) OR ("Chung I Hsueh")) OR ("Chinese Medicine, Traditional")) OR ("Chinese herbal medicine")) OR ("Chinese Herbal Drugs")) OR ("Chinese Drugs, Plant")) OR ("Chinese medicine formulae")) OR ("Chinese medicine formulations")) OR ("Chinese herb")) OR ("Chinese herb therapy")) OR ("Medicine, Traditional")) OR ("Medicine, East Asian Traditional")) OR ("Drugs, Chinese Herbal")) OR ("Herbal Drugs, Chinese")) OR ("herbal medicine")) OR ("herb remedy")) OR ("herb therapy"))) AND (("COVID-19"[MeSH Terms]) OR ((((((((((((((((((((((((((((((((((("COVID 19"[Title/Abstract]) OR ("COVID-19 Virus Disease"[Title/Abstract])) OR ("COVID 19 Virus Disease"[Title/Abstract])) OR ("COVID-19 Virus Diseases"[Title/Abstract])) OR ("Disease, COVID-19 Virus"[Title/Abstract])) OR ("Virus Disease, COVID-19"[Title/Abstract])) OR ("COVID-19 Virus Infection"[Title/Abstract])) OR ("COVID 19 Virus Infection"[Title/Abstract])) OR ("COVID-19 Virus Infections"[Title/Abstract])) OR ("Infection, COVID-19 Virus"[Title/Abstract])) OR ("Virus Infection, COVID-19"[Title/Abstract])) OR ("2019-nCoV Infection"[Title/Abstract])) OR ("2019 nCoV Infection"[Title/Abstract])) OR ("2019-nCoV Infections"[Title/Abstract])) OR ("Infection, 2019-nCoV"[Title/Abstract])) OR ("Coronavirus Disease-19"[Title/Abstract])) OR ("Coronavirus Disease 19"[Title/Abstract])) OR ("2019 Novel Coronavirus Disease"[Title/Abstract])) OR ("2019 Novel Coronavirus Infection"[Title/Abstract])) OR ("2019-nCoV Disease"[Title/Abstract])) OR ("2019 nCoV Disease"[Title/Abstract])) OR ("2019-nCoV Diseases"[Title/Abstract])) OR ("Disease, 2019-nCoV"[Title/Abstract])) OR ("COVID19"[Title/Abstract])) OR ("Coronavirus Disease 2019"[Title/Abstract])) OR ("Disease 2019, Coronavirus"[Title/Abstract])) OR ("SARS Coronavirus 2 Infection"[Title/Abstract])) OR ("SARS-CoV-2 Infection"[Title/Abstract])) OR ("Infection, SARS-CoV-2"[Title/Abstract])) OR ("SARS CoV 2 Infection"[Title/Abstract])) OR ("SARS-CoV-2 Infections"[Title/Abstract])) OR ("COVID-19 Pandemic"[Title/Abstract])) OR ("COVID 19 Pandemic"[Title/Abstract])) OR ("COVID-19 Pandemics"[Title/Abstract])) OR ("Pandemic, COVID-19"[Title/Abstract]))) | 307 |
| (2)Science Direct | #1  Title, abstract, keywords: “coronavirus disease 2019” OR “COVID-19” OR “severe acute respiratory syndrome coronavirus 2” OR “SARS-CoV-2” OR “coronavirus” OR “novel coronavirus”, OR “nCoV”, OR “2019-nCoV”  #2  Title: “Chinese herbal medicine” OR “traditional Chinese medicine” OR “Chinese medicine formulae“ OR “Chinese medicine formulations” OR “Chinese herb” OR “Chinese herb therapy” OR “herbal medicine” OR “herb remedy” OR “herb therapy”  #3  Year: 2019-2021  #1 AND #2 AND #3 | 141 |
| (3)Web of science | #1  TI= ("COVID-19" OR "2019 novel coronavirus disease" OR "COVID19" OR "COVID-19 pandemic" OR "SARS-CoV-2 infection" OR "COVID-19 virus disease" OR "2019 novel coronavirus infection" OR "2019-nCoV infection" OR "coronavirus disease 2019" OR "coronavirus disease-19" OR "2019-nCoV disease" OR "COVID-19 virus infection")  Indexes=SCI-EXPANDED, SSCI, A&HCI, CPCI-S, CPCI-SSH, BKCI-S, BKCI-SSH, ESCI, CCR-EXPANDED, IC Timespan=2019-2021  #2  TS= ("Traditional Chinese Medicine" OR"Traditional Medicine, Chinese" OR"Chinese Traditional Medicine" OR"Chinese Medicine, Traditional" OR"Drugs, Chinese Herbal" OR"Chinese Drugs, Plant" OR"Chinese Herbal Drugs" OR"Herbal Drugs, Chinese" OR"Chinese medicine formulae" OR"Chinese medicine formulations" OR"Chinese herb" OR"Chinese herb therapy" OR"herbal medicine" OR"herb remedy" OR"herb therapy")  Indexes=SCI-EXPANDED, SSCI, A&HCI, CPCI-S, CPCI-SSH, BKCI-S, BKCI-SSH, ESCI, CCR-EXPANDED, IC Timespan=2019-2021  #1 AND #2 | 268 |
| (4)Scopus | TITLE-ABS("2019 novel coronavirus disease" OR "COVID19" OR "COVID-19" OR "COVID-19 virus infection" OR " COVID 19" OR "COVID 2019" OR "COVID-19 pandemic" OR "SARS-CoV-2 infection" OR "COVID-19 virus disease" OR "2019 novel coronavirus infection" OR "2019-nCoV infection" OR "2019-nCoV disease" OR "coronavirus disease 2019" OR "coronavirus disease-19" OR "2019-nCoV disease" OR "nCoV 2019 disease" OR "nCoV 2019 infection" OR "novel coronavirus 2019 disease" OR "novel coronavirus 2019 infection" OR "novel coronavirus disease 2019" OR "novel coronavirus infection 2019" OR "Wuhan coronavirus disease" OR "Wuhan coronavirus infection") AND TITLE-ABS("Traditional Chinese Medicine" OR "Traditional Medicine, Chinese" OR "Traditional Tongue Diagnosis" OR "Tongue Diagnoses, Traditional" OR "Tongue Diagnosis, Traditional" OR "Traditional Tongue Diagnoses" OR "Traditional Tongue Assessment" OR "Tongue Assessment, Traditional" OR "Traditional Tongue Assessments" OR "Hsueh, Chung I" OR "Zhong Yi Xue" OR "Chinese Traditional Medicine" OR "Chung I Hsueh" OR "Chinese Medicine, Traditional" OR "Chinese herbal medicine" OR "Chinese Herbal Drugs" OR "Chinese Drugs, Plant" OR "Chinese medicine formulae" OR "Chinese medicine formulations" OR "Chinese herb" OR "Chinese herb therapy" OR "Medicine, Traditional" OR "Medicine, East Asian Traditional" OR "Drugs, Chinese Herbal" OR "Herbal Drugs, Chinese" OR "herbal medicine" OR "herb remedy" OR "herb therapy") | 464 |
| (5)Google Scholar | (“coronavirus disease 2019” OR “COVID-19” OR “SARS-CoV-2” OR “coronavirus” OR “nCoV”) AND (“Chinese herbal medicine” OR “traditional Chinese medicine” OR “Chinese medicine “ OR “herbal medicine” OR “herb”) | 13,700 |
| (6) Cochrane Library | #1  MeSH descriptor: [Medicine, Chinese Traditional] explode all trees  #2  ("Traditional Chinese Medicine" OR "Traditional Medicine, Chinese" OR "Chinese Traditional Medicine" OR "Chinese Medicine, Traditional" OR "Drugs, Chinese Herbal" OR "Chinese Drugs, Plant" OR "Chinese Herbal Drugs" OR "Herbal Drugs, Chinese" OR "Chinese medicine formulae" OR "Chinese medicine formulations" OR "Chinese herb" OR "Chinese herb therapy" OR "herbal medicine" OR "herb remedy" OR "herb therapy"):ti,ab,kw  #3  #1 or #2  #4  MeSH descriptor: [COVID-19] explode all trees  #5  ("COVID-19" OR "2019 novel coronavirus disease" OR "COVID19" OR "COVID-19 pandemic" OR "SARS-CoV-2 infection" OR "COVID-19 virus disease" OR "2019 novel coronavirus infection" OR "2019-nCoV infection" OR "coronavirus disease 2019" OR "coronavirus disease-19" OR "2019-nCoV disease" OR "COVID-19 virus infection"):ti,ab,kw  #6  #4 OR #5  #7  #3 AND #6 | 65 |
| Embase | ('covid 19':ti OR 'covid 19 virus disease':ti OR 'disease covid 19 virus':ti OR 'virus disease covid 19':ti OR 'covid 19 virus infection':ti OR 'covid-19 virus infections':ti OR 'virus infection covid 19':ti OR '2019 ncov infection':ti OR '2019-ncov infections':ti OR 'infection 2019 ncov':ti OR 'coronavirus disease 19':ti OR '2019 novel coronavirus disease':ti OR '2019 novel coronavirus infection':ti OR '2019 ncov disease':ti OR '2019-ncov diseases':ti OR 'disease 2019 ncov':ti OR 'covid19':ti OR 'coronavirus disease 2019':ti OR 'disease 2019 coronavirus':ti OR 'sars coronavirus 2 infection':ti OR 'infection sars cov 2':ti OR 'sars cov 2 infection':ti OR 'sars-cov-2 infections':ti OR 'covid 19 pandemic':ti OR 'covid-19 pandemics':ti OR 'pandemic covid 19':ti) AND ('traditional chinese medicine':ti,ab,kw OR 'traditional medicine, chinese':ti,ab,kw OR 'chinese traditional medicine':ti,ab,kw OR 'chinese medicine, traditional':ti,ab,kw OR 'drugs, chinese herbal':ti,ab,kw OR 'chinese drugs, plant':ti,ab,kw OR 'chinese herbal drugs':ti,ab,kw OR 'herbal drugs, chinese':ti,ab,kw OR 'chinese medicine formulae':ti,ab,kw OR 'chinese medicine formulations':ti,ab,kw OR 'chinese herb':ti,ab,kw OR 'chinese herb therapy':ti,ab,kw OR 'herbal medicine':ti,ab,kw OR 'herb remedy':ti,ab,kw OR 'herb therapy':ti,ab,kw) AND [2019-2021]/py | 371 |
| (8)ProQuest | ti("coronavirus disease 2019" OR "COVID-19" OR "severe acute respiratory syndrome coronavirus 2" OR "SARS-CoV-2" OR "coronavirus" OR "novel coronavirus" , OR "nCoV" , OR "2019-nCoV") AND su("Chinese herbal medicine" OR "traditional Chinese medicine" OR "Chinese medicine formulae" OR "Chinese medicine formulations" OR "Chinese herb" OR "Chinese herb therapy" OR "herbal medicine" OR "herb remedy" OR "herb therapy") | 429 |
| (9)China National Knowledge Infrastructure Database | #1  (主题=“新型冠状病毒肺炎”) OR (主题=“新冠肺炎”) OR (主题="COVID-19") OR (主题=“2019 冠状病毒病”) OR (主题=“冠状病毒肺炎”)  #2  (主题=“中药”) OR (主题=“中医”) OR (主题=“中西医”) OR (主题=“汤”) OR (主题=“方”) OR (主题=“方剂”) OR (主题=“颗粒”) OR (主题=“合剂”) OR (主题=“胶囊”) OR (主题=“口服液”) OR (主题=“饮”) OR (主题=“注射液”) OR (主题=“丸”) OR (主题=“丹”) OR (主题=“散”) OR (主题=“膏”)  #3  (篇关摘=“临床”) OR (篇关摘=“对照”) OR (篇关摘=“随机”) OR (篇关摘=“观察”) OR (篇关摘=“回顾”) OR (篇关摘=“前瞻”) OR (篇关摘=“队列”)  #1 AND #2 AND #3 | 1,233 |
| (10)Wan Fang database Search strategy | 主题:(“新型冠状病毒肺炎” or “新冠肺炎” or "COVID-19" or “2019 冠状病毒病” or “冠状病毒肺炎”) and 题名或关键词:(“临床” or “对照” or “随机” or “观察” or “回顾” or “前瞻” or “队列” ) and 主题:(“中药” or “中医” or “中西医” or “汤” or “方” or “方剂” or “颗粒” or “合剂” or “胶囊” or “口服液” or “饮”or “注射液”or “丸”or “丹”or “散”or “膏”) | 1,788 |
| (11)Chongqing VIP Chinese Science and Technology Periodical Database (VIP) | #1  题名或关键词=“新型冠状病毒肺炎” OR 题名或关键词=“新冠肺炎” OR 题名或关键词=“COVID 19” OR 题名或关键词=“2019 冠状病毒病” OR 题名或关键词=“冠状病毒肺炎”  #2  任意字段=“中药” OR 任意字段=“中医” OR 任意字段=“中西医” OR 任意字段=“汤” OR 任意字段=“方” OR 任意字段=“方剂” OR 任意字段=“颗粒” OR 任意字段=“合剂” OR任意字段=“胶囊” OR任意字段= “口服液” OR任意字段= “饮” OR任意字段= “注射液” OR任意字段= “丸” OR任意字段= “丹” OR任意字段= “散” OR任意字段= “膏”  #3  任意字段=“临床” OR 任意字段=“对照” OR 任意字段=“随机” OR 任意字段=“观察” OR 任意字段=“回顾” OR 任意字段=“前瞻” OR 任意字段=“队列”  #1 AND #2 AND #3 | 1,903 |
| Overall |  | 20,669 |
